# A Planar Model of an Ankle Joint with Optimized Material Parameters and Hertzian Contact Pairs

**DOI:** 10.3390/ma12162621

**Published:** 2019-08-17

**Authors:** Aleksandra Borucka, Adam Ciszkiewicz

**Affiliations:** Institute of Applied Mechanics, Cracow University of Technology, 31-155 Cracow, Poland

**Keywords:** multibody system, static analysis, stiffness, joint moment, sensitivity analysis

## Abstract

The ankle is one of the most complicated joints in the human body. Its features a plethora of elements with complex behavior. Their functions could be better understood using a planar model of the joint with low parameter count and low numerical complexity. In this study, an accurate planar model of the ankle with optimized material parameters was presented. In order to obtain the model, we proposed an optimizational approach, which fine-tuned the material parameters of two-dimensional links substituting three-dimensional ligaments of the ankle. Furthermore, the cartilage in the model was replaced with Hertzian contact pairs. The model was solved in statics under moment loads up to 5 Nm. The obtained results showed that the structure exhibited angular displacements in the range of the ankle joint and that their range was higher in dorsiflexion than plantarflexion. The structure also displayed a characteristic ramp up of the angular stiffness. The results obtained from the optimized model were in accordance with the experimental results for the ankle. Therefore, the proposed method for fine-tuning the material parameters of its links could be considered viable.

## 1. Introduction

The interest in the mechanics of the human body is ever increasing. Biomechanical models of various complexity are being employed in many industrial and scientific fields, such as surgical simulation [1], presurgical planning [2,3,4], safety systems for vehicles [5], support systems [6], implant design [7], and more. Two main modeling trends can be distinguished in biomechanics: finite element method (FEM) and multibody system method (MBS). In the FEM models, such as in the work of [8], the components of the system are described as deformable structures composed of multiple finite elements. These models tend to describe the structures very accurately, both in terms of geometry and material behavior. Nevertheless, models with large element counts are expensive to solve. Furthermore, because of their complexity and discrete nature, the FEM models can also experience convergence problems. The multibody framework is based on different ideas. Instead of dividing each element of the system into multiple smaller ones, the main elements are substituted with simple mechanical counterparts, such as rigid bodies, springs, cables, dampers, kinematic pairs, Hertzian contact pairs, and more. As a result, the MBS models, such as in the work of [9], are less computationally expensive and easier to analyze. Nevertheless, substituting more complex components of the body joints, such as the contact between the articular surfaces of the bones, is not trivial. This issue is very apparent in many models of the ankle joint.

### 1.1. Modeling the Ankle

The ankle joint is one of the most complex joints in the human body. In fact, the joint itself is composed of multiple smaller joints. In this study, we modeled a part of the ankle referred to as the true ankle joint, which contains two bone segments: the tibia/fibula and the talus/calcaneus. The two segments are connected with several ligaments, which resemble nonlinear cables in their behavior [10]: the anterior tibiotalar ligament (ATT), tibiocalcaneal ligament (TC), posterior tibiotalar ligament (PTT), anterior talofibular ligament (ATF), calcaneofibular ligament (CF), and posterior talofibular ligament (PTF). The bones also interact through a layer of soft tissue called cartilage, which transfers mostly compressive loads. 

As mentioned before, modeling such a complex structure is no easy task. This is why many different models, MBS and FEM, have been presented in literature. These models differ mostly in their description of the cartilage. In the FEM ankle models, the contact of the bones is often described with finite elements, which are deformable [11,12,13,14]. The most common approach in the multibody models is to replace the cartilage with a revolute kinematic pair or two revolute pairs for 3D motions [15,16,17,18,19,20,21]. This constrains the motion of the bones. The ligaments in the mentioned models, both FEM and MBS, are usually substituted with linear or nonlinear flexible cables.

A different method can be seen in the work of [22]. On the basis of experimental results, the authors in [22] have observed that the calcaneofibular and the tibiocalcaneal ligament remain isometric during the passive motion of the ankle. This conclusion has served as a basis for a model presented in the work of [22], in which these ligaments were replaced with rigid links, creating a four-bar linkage model of the ankle. Their model, when compared to the previously-mentioned ones, was composed of only rigid elements. A three-dimensional extension of this methodology has been presented in the literature [23,24,25]. The authors of [24] have idealized the articular surfaces of the talus and the tibia into two sphere-sphere contacts. These contacts were included as constraint equations in their proposed 5-5 parallel ankle model. Similar structures have also been applied in models of different body joints [26,27,28,29,30]. In another paper [31], the articular surfaces of the bones have been digitized and represented as a triangular surface mesh forming a structure with multiple contact points.

Regardless of the ankle model, the aforementioned multibody cartilage substitutes are kinematic in their nature—the bones and their cartilage, while allowed to roll or slide and roll, can never deform. This issue has been addressed in another paper [32], in which the authors have proposed an extended 5-5 parallel model with deformable contact pairs. Nevertheless, the equation used to compute the forces acting on the contact pairs has not been based on an established contact model. A different method has been utilized for the knee joint in the works of [33,34]. In these studies, the contact forces in the cartilage were computed using Hertz contact theory [35]. This simplified the behavior of the cartilage to linear elastic, but offered low computational complexity when compared with FEM models.

### 1.2. Dimensionality of the Models

Another distinction within the multibody models of the human body joints can be observed in their dimensionality. The models can be either three-dimensional or two-dimensional. The three-dimensional ones are capable of reproducing all of the allowed motions in the joint. Nevertheless, they are likely to experience convergence problems, have larger number of parameters, and are expensive to solve. On the other hand, the two-dimensional models can be used to study the main motion within the joint with a very low parameter count and numerical complexity. Despite these advantages, there is no easy way to prepare a two-dimensional model of a three-dimensional body joint. This is especially evident when assigning the material parameters to the elements of the model, such as the ligaments or the cartilage. Most research groups utilize the experimental results published in literature [34,36]. Nevertheless, for certain body joints, this approach can lead to significant inaccuracies within the results. It is thus advisable to establish a reusable framework for obtaining material parameters in two-dimensional joint models. This issue is further explored in Section 2.3 of the paper.

### 1.3. The Aim of the Study and Its Novel Aspects

The main aim of this study was to present an accurate planar model of the ankle with optimized material parameters. In order to obtain a planar model of the three-dimensional joint, we proposed an optimizational approach to fine-tune the material parameters of two-dimensional links substituting three-dimensional ligaments of the ankle. Furthermore, we combined the observations from the work of [24] and the method presented in the work of [34] to substitute the cartilage in the ankle with Hertzian contact pairs. 

The presented model contained two rigid bodies corresponding to the bones; six nonlinear cables, which substituted the ligaments; and two Hertzian contact pairs, which represented the cartilage. To substitute the ligaments with two-dimensional cables, we applied our custom optimization procedure based on nonlinear least squares, which fine-tuned the material parameters of the links. The obtained model was solved for plantarflexion and dorsiflexion in statics and compared with experimental and computational results available in the literature. The obtained results showed that the proposed structure with optimized material parameters exhibited similar behavior to that of the ankle joint. Finally, an additional sensitivity analysis was performed. 

Our main contributions to the existing research were in proposing a novel approach to substitute three-dimensional ligaments with optimal two-dimensional cables and in modeling the cartilage interaction in the ankle with two Hertzian contact pairs. 

There were two major advantages of the presented contributions. Firstly, the methodology for optimizing two-dimensional cables allowed us to obtain an accurate two-dimensional model of the three-dimensional ankle joint. This optimized model could be used to study the load states of the ligaments in the joint. Secondly, the cartilage modeled with Hertzian contact pairs had a more physical response when compared with the typically used ball-socket type constraints. It allowed for the deformation of the tissue, while its computational cost was negligible. To the best of our knowledge, such a model and methodology have never been considered for the ankle joint. The following sections contain a detailed description of the model, the method, and the obtained results.

## 2. Materials and Methods

### 2.1. The Model

In order to prepare the model of the ankle, we assumed that the tibia and the fibula were rigidly connected and formed one rigid body referred to as the basis - tibia-fibula segment (TFS). The talus and the calcaneus were also treated as one rigid body and referred to as the moving body - talus-calcaneus segment (TCS). In the model TFS was fixed, while TCS could be displaced. Both segments had their own separate reference frames. They were also connected by a system of six ligaments and two contact pairs. The ligaments were substituted with nonlinear cables, while the cartilage interaction was idealized into two sphere–sphere Hertzian contact pairs.

**Figure 1:**
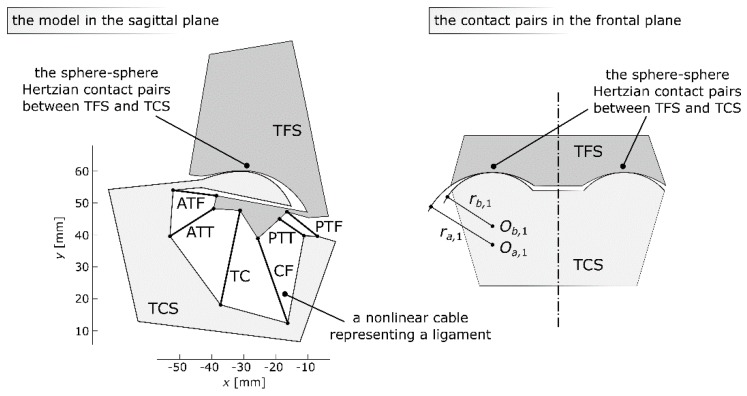
The proposed planar model of the ankle joint in the sagittal plane and a schematic representation of its spherical contact pairs in the frontal plane. TFS, basis-tibia-fibula segment; TCS, moving body-talus-calcaneus segment; ATT, anterior tibiotalar ligament; TC, tibiocalcaneal ligament; PTT, posterior tibiotalar ligament; ATF, anterior talofibular ligament; CF, calcaneofibular ligament; PTF, posterior talofibular ligament.

To summarize, the presented model contained the following (see Figure 1):

1. Two rigid bodies: the basis - tibia-fibula segment (TFS), the moving body - talus-calcaneus segment (TCS);
2. Six nonlinear planar cables, representing ATT, TC, PTT, ATF, CF, and PTF;
3. Two symmetrical sphere–sphere Hertzian contact pairs representing the cartilage between the tibia and the talus;
4. To specify the location of the TFS with regard to the TCS, three variables were used;
5. One angular coordinate *θ*, used to compute the rotation matrix ***R*** from the TCS to TFS:
  (1) $$R\left(\theta \right)=\left[\begin{array}{cc} \cos\theta & -\sin\theta \\ \sin\theta & \cos\theta \end{array}\right];$$
6. Two linear coordinates, which formed the position vector between the frames ***p***:
  (2) $$p={\left[\begin{array}{cc} p_{x} & p_{y} \end{array}\right]}^{T}.$$

### 2.2. The Ligaments

As mentioned before, the ligaments in the presented model were substituted with two-dimensional, nonlinear cables. This section contains the equations necessary to compute the loads generated by the cables. The procedure used to optimize the material parameters of the cables is described in the next section.

The first step in computing the cable force and moment was to calculate its length. Given the relative location of the TCS with regards to the TFS specified by *θ* and ***p***, the length *l_i_* of the cable at the location was obtained as follows:

(3) $$l_{i}=\left|b_{i}-a_{i}\right|,$$

where ***a_i_*** (***b_i_***)—the position vector of the *i*-th cable’s attachment to the TCS (TFS), *i* ∈ {ATF, ATT, TC, CF, PTT, PTF}.

Note that the vectors in (3) were expressed in the reference frame of TFS. As the attachments to the TCS were given in its local reference frame, it was necessary to apply the following transformation:

(4) $$b_{i}=R\left(\theta \right)b_{i}^{TCS}+p,$$

where $b_{i}^{TCS}$—the position vector of the *i*-th cable’s attachment to the TCS in the TCS reference frame, ***R***—the rotation matrix from the TCS to TFS, and ***p***—the position vector between the frames.

In the next step, the unit vector for the direction of the cable force was obtained:

(5) $$F_{i}^{o}=\frac{b_{i}-a_{i}}{l_{i}}.$$

The value of the cable force was computed using an exponential model [37]:

(6) $$F_{i}=A_{i}\exp\left(B_{i}\varepsilon_{i}-1\right),$$

where *A_i_*, *B_i_*—the stiffness coefficients of the ligament *i*, *ε_i_*—the strain of the ligament *i*:

(7) $$\varepsilon_{i}=\frac{l_{i}-l_{slack,i}}{l_{slack,i}},$$

where *l_slack,i_*—the slack length of the ligament *i* (computed at the neutral location of the TCS).

Finally, the force vector of the ligament was obtained by multiplying the direction vector by its corresponding force value *F_i_*:

(8) $$F_{i}=\{\begin{array}{ll} -F_{i}^{o}F_{i}, & \mathrm{for}\varepsilon_{i}>0\\ 0, & \mathrm{for}\varepsilon_{i}\leq 0 \end{array}.$$

Each ligament also generated a moment, which was computed as follows:

(9) $$M_{i}=b_{i}\times F_{i}.$$

It is worth mentioning that the presented vector equations are valid also in three-dimensional problems.

### 2.3. Optimizing the Material Parameters of The Ligaments

**Figure 2:**
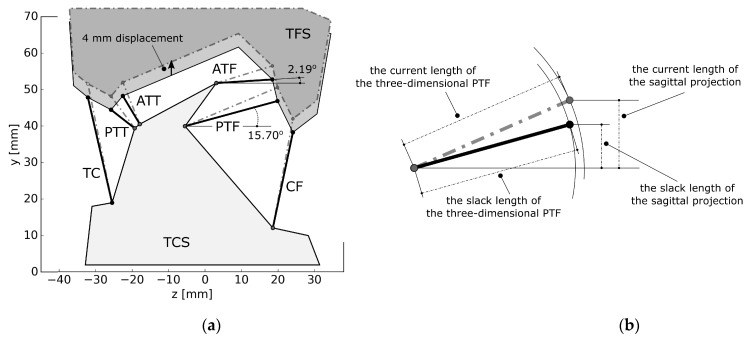
(**a**) The ligament system of the ankle as seen in the frontal plane in two configurations: initial and with the segments displaced by 4 mm along the y-axis; (**b**) PTF and its projection onto the sagittal plane in two configurations: initial and with the segments displaced by 4 mm along the y-axis.

In the actual ankle, the ligaments are distributed spatially. The proposed model contains only two-dimensional cables. This simplification can create significant errors when computing the strains of the ligaments. Figure 2a presents a ligament system of an ankle as seen in the frontal plane. Two of the ligaments connecting the fibula and the talus, PTF and ATF, are nearly horizontal, with their angles measured with regards to the z-axis at 15.70° and 2.19°, respectively (see Figure 2a). As their sagittal projections have very low slack lengths, the strains of their three-dimensional representations are much lower than the strains of the sagittal substitutes—see Figure 2b. Furthermore, the forces generated by the three-dimensional ligaments are also three-dimensional—their force vectors have to be projected onto the sagittal plane. This creates a highly non-linear relationship between the three-dimensional ligaments and their planar substitutes. Therefore, the material parameters for the links in the planar model cannot be obtained directly from experiments. 

**Figure 3:**
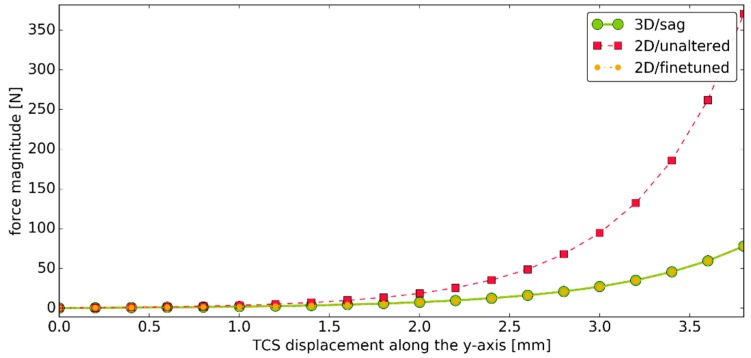
The graph of one of the considered displacements versus the value of the force generated by the following: a three-dimensional PTT ligament with unaltered stiffness parameters from the work of [37] in the sagittal plane (green), its planar counterpart with unaltered stiffness parameters from the work of [37] (red), and a planar counterpart with fine-tuned material parameters using the proposed custom approach (yellow).

To further visualize this issue, Figure 3 presents the force generated by a three-dimensional PTT ligament and its planar counterpart when both operate under the unaltered stiffness parameters from experiments [37]. The graph also contains additional results for comparison obtained with our custom procedure, which fine-tuned the material parameters of the two-dimensional substitutes. 

Our fine-tuning method was as follows. Firstly, based on two projections of the ankle available in medical atlases, we created a three-dimensional model of the ankle’s ligament system in the unloaded configuration. The three-dimensional model was then projected on the sagittal plane to create its planar representation, used in subsequent simulations. Then, we displaced the segments by ±4 mm along the y-axis and the x-axis. In total, four displacements were considered—one of them can be seen in Figure 2a. The value of the displacements—4 mm—was not arbitrary. In our three-dimensional model, under such displacements, the mean value of the peak forces generated by the ligaments was at 374.92 N. Furthermore, all ligaments were active. Smaller displacements resulted in PTF generating negligible forces.

For each displacement, two quantities were computed for every ligament: the force generated by a three-dimensional link ***F_3D_***—using Equation (8)—and the strain of its planar counterpart *ε*_2*D*_—using Equation (7). The three-dimensional force ***F_3D_*** was then projected onto the sagittal plane and the magnitude of this projection *F_3D/sag_* was obtained. Finally, we assumed that the planar substitute under *ε*_2*D*_ should generate the force of equal value to the one generated by the three-dimensional link when projected onto sagittal plane *F_3D/sag_* under all of the considered displacements. This allowed us to formulate the following set of residual equations:

(10) $$\{\begin{array}{c} res_{1}=A_{i}\exp\left(B_{i}\varepsilon_{2D/disp1}-1\right)-F_{3D/sag/disp1}\\ res_{2}=A_{i}\exp\left(B_{i}\varepsilon_{2D/disp2}-1\right)-F_{3D/sag/disp2}\\ res_{3}=A_{i}\exp\left(B_{i}\varepsilon_{2D/disp3}-1\right)-F_{3D/sag/disp3}\\ res_{4}=A_{i}\exp\left(B_{i}\varepsilon_{2D/disp4}-1\right)-F_{3D/sag/disp4} \end{array},$$

where *A_i_*, *B_i_*—the stiffness coefficients of the planar cable *i* representing ligament *i*, *ε*_2*D/disp j*_—the strain of the planar cable *i* while the segments are under displacement *j*, and *F_3D/sag/disp j_*—the force value generated by the three-dimensional ligament *i* when projected onto sagittal plane while the segments are under displacement *j*.

To find the optimal values of the two material parameters in system (10), we employed nonlinear least squares with *curve_fit* function implemented in Scipy. The optimization was performed for each link of the planar model and the computed stiffness parameters were used in subsequent simulations. This allowed us to obtain accurate two-dimensional representations of three-dimensional ligaments. The main advantage of this approach was that it modified only the material parameters of the links. Their geometry was unaltered. This means that the geometrical parameters of the model could be obtained directly from a planar or a spatial scan of the ankle.

### 2.4. The Cartilage

In the ankle joint, the bones interact through a deformable soft-tissue layer called cartilage. In the proposed model, this cartilage was modeled using the Hertz contact theory as a linear elastic material. A similar approach has been proposed in the work of [34] for the knee joint. As shown in Figure 1, the model contained two sphere–sphere contact pairs, parallel in the frontal plane—this structure was chosen based on the results from the work of [24]. The value of the contact force between the contacting elements was computed using the following equation [34]:

(11) $$F_{c,i}=K_{i}\delta_{i}^{3/2},$$

where *F_c,i_*—the value of the contact force generated by the contact pair *i*, *K_i_*—the generalized stiffness coefficient for the contact pair *i*, and *δ_i_*—the relative penetration of the bodies in the contact pair *i*.

The generalized stiffness for the sphere–sphere contact, considering female and male spheres employed in the model, was obtained as per the following [34]:

(12) $$K_{i}=\frac{2E_{i}}{3(1-\nu_{i}^{2})}\sqrt{\frac{r_{a,i}r_{b,i}}{r_{a,i}-r_{b,i}}},$$

where *r_a,i_*, *r_b,i_*—the radii of the spheres in the contact pair *i* (the radii were assumed to be of positive values for the male and the female sphere), *ν_i_*—Poisson’s ratio for the elements in the contact pair *i*, and *E_i_*—Young’s modulus for the elements in the contact pair *i* (both spheres in the contact pairs were assumed to be of the same material).

The relative penetration *δ_i_* of the spheres was obtained as follows:

(13) $$\delta_{i}=\left|O_{b,i}-O_{a,i}\right|+r_{b,i}-r_{a,i},$$

where ***O_a,i_***, ***O_b,i_*** —the position vectors of sphere centers with regard to the TFS frame.

The vector ***O_b,i_*** had to be obtained through a reference frame transformation:

(14) $$O_{b,i}=R\left(\theta \right)O_{b,i}^{TCS}+p.$$

The unit vector for the direction of the contact force was computed using the following:

(15) $$F_{c,i}^{o}=\frac{O_{b,i}-O_{a,i}}{\left|O_{b,i}-O_{a,i}\right|}.$$

Finally, the contact force between the spheres was obtained:

(16) $$F_{c,i}=\{\begin{array}{ll} -F_{c,i}^{o}F_{c,i}, & \mathrm{for}\delta_{i}>0\\ 0, & \mathrm{for}\delta_{i}\leq 0 \end{array}.$$

The contact force’s attachment to the TCS was assumed to be at the half of the penetration:

(17) $$b_{c,i}=O_{b,i}+F_{c,i}^{o}\left(r_{b,i}+\delta_{i}/2\right).$$

Its moment was computed as follows:

(18) $$M_{c,i}=b_{c,i}\times F_{c,i}.$$

### 2.5. Solving Elastostatic Problems

The proposed model was subjected to static loading conditions. The problem we solved was formulated as follows: Given a loading condition (i.e., external forces and moments acting on the TCS), find a location of the TCS (i.e., one angular coordinate *θ*, which specifies the rotation matrix ***R***, and two linear coordinates that form the position vector ***p***), in which the sums of the forces and the moments acting on the TCS are equal to 0. This condition was specified as the following equilibrium equations [38,39]:

(19) $$\left\{ \begin{array}{l} \sum_{i=1}^{n}F_{i}+\sum_{j=1}^{m}F_{c,j}+F_{ext}=0\\ \sum_{i=1}^{n}M_{i}+\sum_{j=1}^{m}M_{c,j}+M_{ext}=0 \end{array}\right.,$$

where ***F_i_*** (***M_i_***)—the forces (moments) generated by the nonlinear cables representing the ligaments, ***F_c,i_*** (***M_c,i_***)—the forces (the moments) generated by the spheres in contact, ***F_ext_*** (***M_ext_***)—the external force (moment) acting on the TCS, and *n* (*m*)—the number of the cables (contact pairs). 

Equation (3) was solved in Python with a least squares approach using the Levenberg–Marquardt method from Scipy library [40]. The solution was accepted if the sum of the residual loads (***F_x_***, ***F_y_*** and ***M***) was less than 1.0 × 10^−10^.

## 3. Results

### 3.1. The Input Dataset

The geometries of the ligament system and the contact pairs, as presented in Figure 1, were based on various anatomical atlases and published ankle models, primarily [41]. The model was assumed to be unloaded in the presented configuration. The slack lengths of the ligaments were computed based on the location of the TCS, as presented in Figure 10. The initial stiffness parameters for the ligaments were assumed after the work of [37], while the Young‘s modulus and the Poisson’s ratio for the Hertzian contact pairs were set to 10.00 MPa and 0.46, respectively. The two contact pairs were assumed to be symmetrical. The stiffness parameters for the cables were optimized with the procedure described in Section 2.3. The model with the optimized material parameters was solved iteratively in plantarflexion and dorsiflexion for the following moment loads:

- Simulation #1: ***M_ext_*** = −0.20:0.20 Nm in 51 steps;
- Simulation #2: ***M_ext_*** = −5.00:5.00 Nm in 51 steps;
- Simulation #3: ***M_ext_*** = −5.00:5.00 Nm in 51 steps the geometry of PTT was modified to assess the sensitivity of the model.

The negative values of the external moment ***M_ext_*** corresponded to dorsiflexion. Conversely, the positively-valued moments caused plantarflexion.

### 3.2. Simulation #1

**Figure 4:**
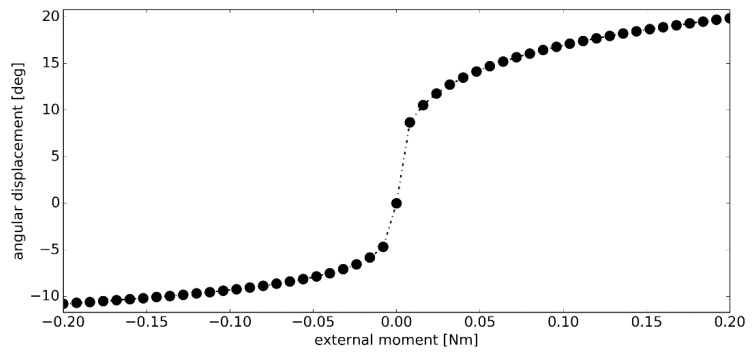
The angular displacement *Δθ* versus the external moment ***M_ext_*** in simulation #1.

The angular displacements in dorsiflexion and plantarflexion caused by the external moment ***M_ext_*** were presented in Figure 4. In both cases, the displacement function was highly nonlinear. An initial low-stiffness region was followed by a significant increase in stiffness in the latter part of the simulation. The total angular displacement of the model, under the assumed loading conditions, was 30.65 deg–10.79 deg in dorsiflexion and 19.86 deg in plantarflexion.

**Figure 5:**
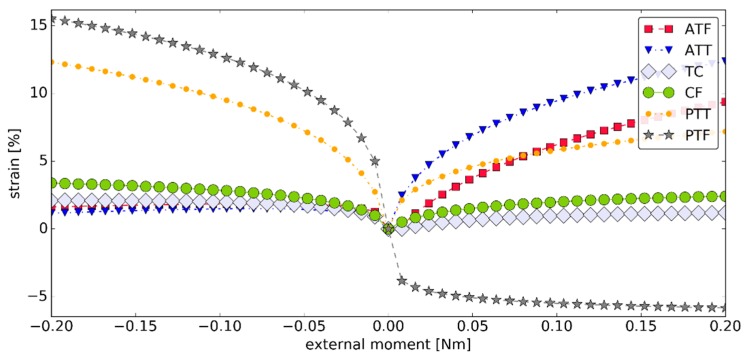
The strains within the ligaments as seen in the sagittal plane versus the external moment ***M_ext_*** in simulation #1 (negative [positive] strain corresponded to an inactive [active] ligament).

As with the angular displacement, significant nonlinearity was observed in the strain function for the ligaments—see Figure 5. The largest increase of strain for all ligaments occurred in the initial part of the simulation. ATT was highly active during plantarflexion. TC and CF remained fairly isometric during the simulation. The peak strain for TC and CF was 2.09% and 3.38%, respectively, with the mean strain after modulus of 2.08% and 2.22%, respectively.

**Figure 6:**
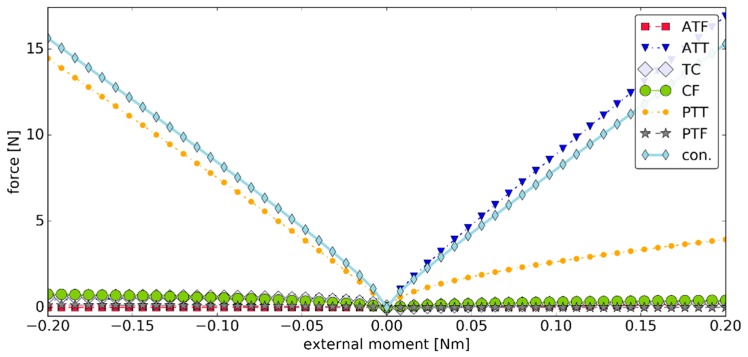
The values of the forces generated by the ligaments versus the external moment ***M_ext_*** in simulation #1, where "con." is the magnitude of the contact force generated by the two contact pairs.

The values of the forces generated by the ligaments during simulation #1 were presented in Figure 6. In this simulation, TC and CF had little contribution to the load state of the joint. This was because of the assumed exponential model for the ligaments, in which low strains corresponded to forces of very small magnitudes. The highest ligament force was observed for ATT in plantarflexion and PTT in dorsiflexion. Their values were under 17.00 N. The contact pairs were active through the simulation exhibiting a fairly linear contact force ramp up in both plantarflexion and dorsiflexion with forces up to 16.91 N.

### 3.3. Simulation #2

**Figure 7:**
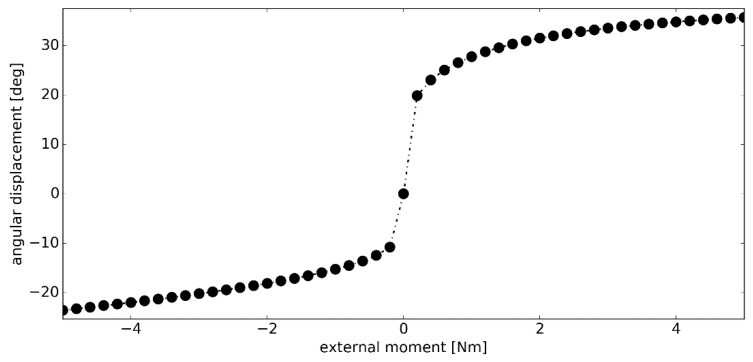
The angular displacement *Δθ* versus the external moment ***M_ext_*** in simulation #2.

In the second simulation, the magnitude of the external moment ***M_ext_*** was increased to 5.00 Nm. As seen in Figure 7, after 0.20 Nm, the angular stiffness of the model continued to increase. Again, higher stiffness was observed in dorsiflexion, which resulted in a significantly smaller range of motion in this phase of the simulation. The total displacement of the model, under the assumed loading conditions, was 59.24 deg–23.54 deg in dorsiflexion and 35.70 deg in plantarflexion. 

**Figure 8:**
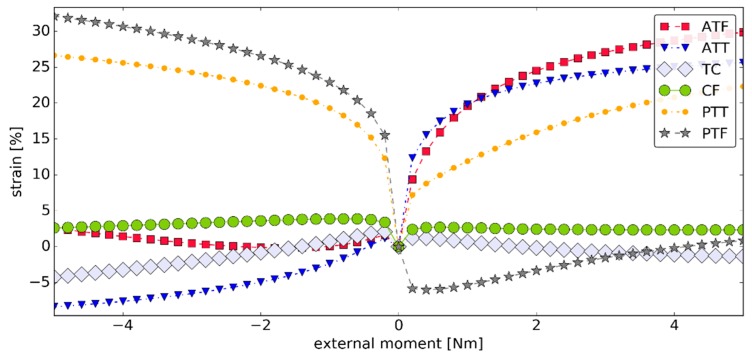
The strains within the ligaments as seen in the sagittal plane versus the external moment ***M_ext_*** in simulation #2 (negative [positive] strain corresponded to an inactive [active] ligament).

Simulation #2 highlighted the isometric behavior of both TC and CF. The peak strain for TC and CF was 4.25% and 3.89%, respectively, with the mean strain after modulus of 1.38% and 2.83%, respectively. While these values were higher than in the previous simulation, they remained negligible when compared with the strains of the other ligaments—see Figure 8.

**Figure 9:**
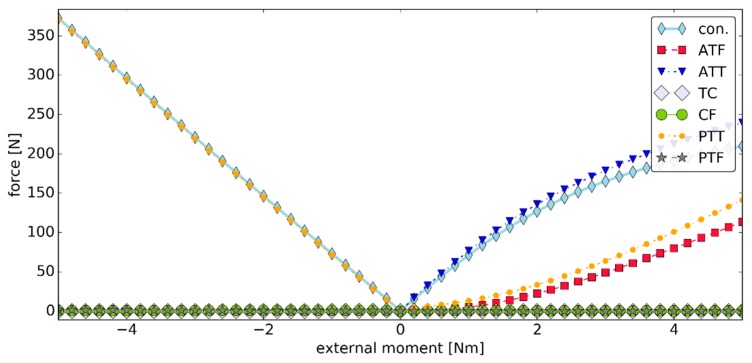
The values of the forces generated by the ligaments versus the external moment ***M_ext_*** in simulation #2, where "con." is the magnitude of the contact force generated by the two contact pairs.

As seen in Figure 9, the forces generated by the ligaments were below 375.00 N. The highest force value of 370.93 N was observed for PTT in dorsiflexion. Again, TC and CF generated negligible forces throughout the simulation. During plantarflexion, the model was mostly stabilized by ATT, which generated forces up to 239.78 The contact pairs were more active during dorsiflexion, with a contact force of magnitude up to 372.62 N.

### 3.4. Simulation #3—Elements of a Sensitivity Analysis

**Figure 10:**
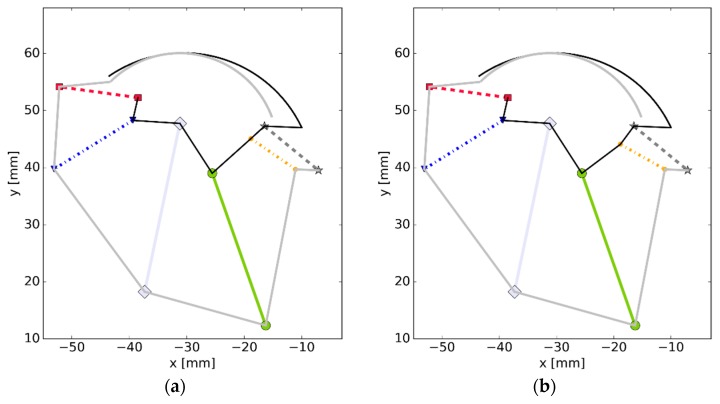
The model (as seen in the prepared software) (**a**) used in simulations #1 and #2; (**b**) with a modified PTT, where the grey (black) platform corresponds to TCS (TFS) and the colors of the links correspond to the ones used in previous figures.

In order to perform a preliminary assessment of how sensitive the presented model was to parameter changes, we decided to modify the geometry of PTT. In this case, only 1 mm was added to the *y* coordinate of the PTT’s attachment to TFS—see Figure 10. 

**Figure 11:**
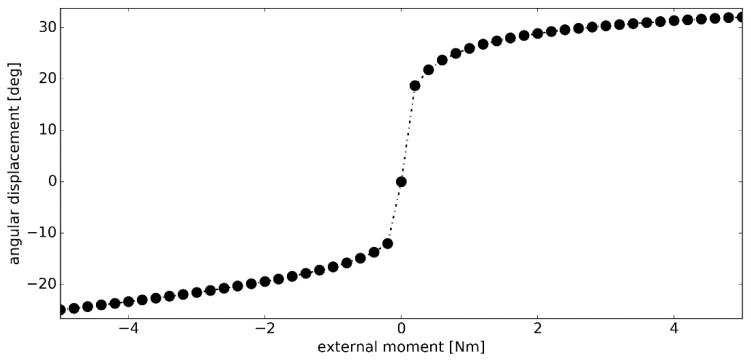
The angular displacement *Δθ* versus the external moment ***M_ext_*** in simulation #3.

The change in the PTT’s attachment resulted in a slightly increased range in the dorsiflexion, from 23.54 deg to 24.94 deg, and a decrease in plantarflexion range, from 35.70 deg to 32.03 deg—see Figure 11. The total range was at 56.97 deg.

**Figure 12:**
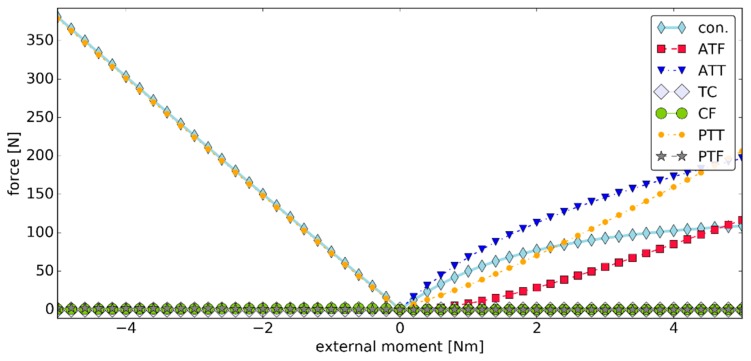
The values of the forces generated by the ligaments versus the external moment ***M_ext_*** in simulation #3, where "con." is the magnitude of the contact force generated by the two contact pairs.

While the changes in the displacement ranges could be considered negligible, the load state in the model was significantly affected. As seen in Figure 12, in this simulation, both ATT and the contact pairs generated forces of much lower magnitudes in plantarflexion when compared with the previous simulation. In the case of the contact pairs, the decrease in the force value was close to 100.00 N. To compensate for these changes, higher forces were generated in PTT and ATF during this phase of the simulation. The isometric behavior of TC and CF was still observed after the parameter modification.

## 4. Discussion

The proposed model with the optimized material parameters exhibited angular displacements in the range comparable to that of the actual ankle joint [42]. Furthermore, the range of the angular displacements was higher in plantarflexion than dorsiflexion, as noted in the literature [17,24,42]. A characteristic nonlinear ramp up of the angular stiffness was also observed. The overall shape of the angular displacement versus the external moment closely resembled that reported in other studies [17,42]. 

The forces generated by the cables never exceeded 375.00 N. Judging by the experimental studies performed on the ankle ligaments [37,42], the obtained values were within their safe limits. CF and TC experienced low peak and mean strains in all simulations. This showed their isometric and was in fair agreement with experimental studies on the ankle joint’s passive motion and its kinematic models [22,23,24,25]. ATT remained active through plantarflexion, while PTT contributed mostly in dorsiflexion. Similar behavior of ATT was observed in an experimental study performed with magnetic resonance [43]. Two of the ligaments connecting the fibula and the talus—PTF and ATF—had little contribution to the load state of the joint. This could be explained by their nearly-horizontal projection in the frontal plane—these ligaments experience low strains during displacements in the sagittal plane. This result validated the proposed ligament fine-tuning procedure. 

The preliminary sensitivity analysis highlighted the high sensitivity of the model to its geometric parameters. A small change in the PTT’s geometry significantly impacted the load state in the model. This result suggests that the ligament behavior in the models of joints with flexible links is highly correlated with the geometrical parameters of the model. A more comprehensive analysis of the model sensitivity should be carried out in the future.

As the results obtained from the model were in accordance with the experimental results for the ankle, the proposed methodology for fine-tuning the material parameters of the two-dimensional links was viable. In some cases (Figure 3), the fine-tuned response was nearly identical to the one from the ligament. Nevertheless, our initial extended tests suggest that for some displacements of the segments, the geometrical and material nonlinearities cannot be fully captured by the assumed exponential model. In future work, more complex models for the force–strain relationship, along with more displacements, should be considered. Furthermore, it may also be beneficial to consider different displacements for each of the ligaments. This modification may require employing complex optimization procedures, such as the widely applied genetic algorithm [3,44,45,46].

## 5. Conclusions

In this study, a novel optimizational approach to fine-tune the material parameters of two-dimensional links substituting three-dimensional ligaments in the ankle was proposed. The approach was applied to the true ankle joint to obtain an accurate planar model of the ankle. The model consisted of two rigid bodies; six nonlinear cables representing the ligaments; and two sphere–sphere Hertzian contact pairs, which substituted the cartilage. The second novelty of the study was in the cartilage description. The proposed model allowed for the deformation of the cartilage, whereas most multibody models of the ankle substitute the articular surfaces using various forms of constraints. In this implementation, the motion of the bones in the ankle was unconstrained—the contact was only active if the cartilage of the bones experienced relative penetration. The model was successfully verified against varied experimental and computational results published in the literature. Its possible applications include presurgical planning, rehabilitation planning, and design of artificial joints. Because of its simplicity, the model can also be employed in education regarding the functions of the ankle joint’s elements. 

## References

[1] Kudasik T., Libura M., Markowska O., Miechowicz S. (2015). Methods for designing and fabrication large-size medical models for orthopaedics. Bull. Polish Acad. Sci. Tech. Sci..

[2] Pietruski P., Majak M., Światek-Najwer E., Popek M., Jaworowski J., Zuk M., Nowakowski F. (2015). Image-guided bone resection as a prospective alternative to cutting templates-A preliminary study. J. Craniomaxillofac. Surg..

[3] Ciszkiewicz A., Milewski G. (2018). Path planning for minimally-invasive knee surgery using a hybrid optimization procedure. Comput. Methods Biomech. Biomed. Engin..

[4] Ciszkiewicz A., Lorkowski J., Milewski G. (2018). A novel planning solution for semi-autonomous aspiration of Baker’s cysts. Int. J. Med. Robot..

[5] Baranowski P., Buka J., Damaziak K., Małachowski J., Mazurkiewicz Ł., Muszyński A. (2016). Numerical analysis of child restraint system equipped with built-in belts pretensioner during frontal impact. Proceedings of the Springer Proceedings in Mathematics & Statistics.

[6] Clin J., Aubin C.É., Parent S., Labelle H. (2011). Biomechanical modeling of brace treatment of scoliosis: Effects of gravitational loads. Med. Biol. Eng. Comput..

[7] Szepietowska K., Magnain B., Lubowiecka I., Florentin E. (2018). Sensitivity analysis based on non-intrusive regression-based polynomial chaos expansion for surgical mesh modelling. Struct. Multidiscip. Optim..

[8] Szkoda-Poliszuk K., Żak M., Pezowicz C. (2018). Finite element analysis of the influence of three-joint spinal complex on the change of the intervertebral disc bulge and height. Int. J. Numer. Method. Biomed. Eng..

[9] Sherman M.A., Seth A., Delp S.L. (2011). Simbody: Multibody dynamics for biomedical research. Proc. IUTAM.

[10] Sensini A., Cristofolini L. (2018). Biofabrication of Electrospun Scaffolds for the Regeneration of Tendons and Ligaments. Materials.

[11] Beaugonin M., Haug E., Cesari D. (1997). Improvement of numerical ankle/foot model: modeling of deformable bone. SAE Trans..

[12] Klekiel T., Będziński R. (2015). Finite element analysis of large deformation of articular cartilage in upper ankle joint of occupant in military vehicles during explosion. Arch. Metall. Mater..

[13] Cheung T.M.J., Zhang M., An K.N. (2004). Effects of plantar fascia stiffness on the biomechanical responses of the ankle-foot complex. Clin. Biomech..

[14] Tannous R.E., Bandak F.A., Toridis T.G., Eppinger R.H. A Three-Dimensional finite element model of the human ankle: Development and preliminary application to axial impulsive loading. Proceedings of the 40th Stapp Car Crash Conference.

[15] Delp S.L., Anderson F.C., Arnold A.S., Loan P., Habib A., John C.T., Guendelman E., Thelen D.G. (2007). OpenSim: Open-source software to create and analyze dynamic simulations of movement. IEEE Trans. Biomed. Eng..

[16] Dettwyler M., Stacoff A., Kramers-De Quervain I.A., Stüssi E. (2004). Modelling of the ankle joint complex. Reflections with regards to ankle prostheses. Foot Ankle Surg..

[17] Jamwal P.K., Hussain S., Tsoi Y.H., Ghayesh M.H., Xie S.Q. (2017). Musculoskeletal modelling of human ankle complex: Estimation of ankle joint moments. Clin. Biomech..

[18] Lewis G.S., Sommer H.J., Piazza S.J. (2006). In Vitro assessment of a motion-based optimization method for locating the talocrural and subtalar joint axes. J. Biomech. Eng..

[19] Montefiori E., Modenese L., Di Marco R., Magni-Manzoni S., Malattia C., Petrarca M., Ronchetti A., de Horatio L.T., van Dijkhuizen P., Wang A. (2019). An image-based kinematic model of the tibiotalar and subtalar joints and its application to gait analysis in children with Juvenile Idiopathic Arthritis. J. Biomech..

[20] Vandenbogert A., Smith G. (1994). In vivo determination of anatomical axes of ankle joint complex. J. Biomech..

[21] Wright I.C., Bogert A.J. (2000). Van Den The influence of foot position on ankle sprain. J. Biomech..

[22] Leardini A., O’Connor J.J., Catani F., Giannini S. (1999). A geometric model of the human ankle joint. J. Biomech..

[23] Baldisserri B. (2012). New mechanisms for modelling the motion of the human ankle complex. Ph.D. Thesis.

[24] Franci R., Parenti-Castelli V. A 5-5 one-degree-of-freedom fully parallel mechanism for the modeling of passive motion at the human ankle joint. Proceedings of the ASME 2007 International Design Engineering Technical Conferences & Computers and Information in Engineering Conference IDETC/CIE 2007.

[25] Gregorio R., Parenti-Castelli V., O’Connor J.J., Leardini A. (2007). Mathematical models of passive motion at the human ankle joint by equivalent spatial parallel mechanisms. Med. Biol. Eng. Comput..

[26] Ciszkiewicz A., Milewski G. (2016). A novel kinematic model for a functional spinal unit and a lumbar spine. Acta Bioeng. Biomech..

[27] Ottoboni A., Parenti-Castelli V., Sancisi N., Belvedere C., Leardini A. (2010). Articular surface approximation in equivalent spatial parallel mechanism models of the human knee joint: An experiment-based assessment. Proc. Inst. Mech. Eng. H..

[28] Sancisi N., Parenti-Castelli V. (2011). A novel 3D parallel mechanism for the passive motion simulation of the patella-femur-tibia complex. Meccanica.

[29] Sancisi N., Parenti-Castelli V. (2010). A 1-Dof parallel spherical wrist for the modelling of the knee passive motion. Mech. Mach. Theory.

[30] Ciszkiewicz A., Knapczyk J. (2016). Load analysis of a patellofemoral joint by a quadriceps muscle. Acta Bioeng. Biomech..

[31] Liacouras P.C., Wayne J.S. (2007). Computational modeling to predict mechanical function of joints: application to the lower leg with simulation of two cadaver studies. J. Biomech. Eng..

[32] Forlani M., Sancisi N., Parenti-Castelli V. (2015). A Three-Dimensional Ankle Kinetostatic Model to Simulate Loaded and Unloaded Joint Motion. J. Biomech. Eng..

[33] Machado M., Moreira P., Flores P., Lankarani H.M. (2012). Compliant contact force models in multibody dynamics: Evolution of the Hertz contact theory. Mech. Mach. Theory.

[34] Machado M., Flores P., Claro J.C.P., Ambrósio J., Silva M., Completo A., Lankarani H.M. (2009). Development of a planar multibody model of the human knee joint. Nonlinear Dyn..

[35] Hertz H. (1896). On the contact of solids—on the contact of rigid elastic solids and on hardness. Miscellaneous Papers.

[36] Moeinzadeh M.H., Engin A.E., Akkas N. (1983). Two-dimensional dynamic modelling of human knee joint. J. Biomech..

[37] Funk J.R., Hall G.W., Crandall J.R., Pilkey W.D. (2002). Linear and Quasi-Linear Viscoelastic Characterization of Ankle Ligaments. J. Biomech. Eng..

[38] Ciszkiewicz A., Milewski G. (2018). Ligament-based spine-segment mechanisms. Bull. Polish Acad. Sci. Tech. Sci..

[39] Ciszkiewicz A., Milewski G. (2019). Structural and material optimization for automatic synthesis of spine-segment mechanisms for humanoid robots with custom sti ff ness profiles. Materials.

[40] Van der Walt S., Colbert S.C., Varoquaux G. (2011). The numpy array: A structure for efficient numerical computation. Comput. Sci. Eng..

[41] Standring S. (2005). Gray’s Anatomy: The Anatomical Basis of Medicine and Surgery.

[42] Chen J., Siegler S., Schneck C.D. (1988). The Three-dimensional kinematics and flexibility characteristics of the human ankle and subtalar joint—Part II: Flexibility characteristics. J. Biomech. Eng..

[43] De Asla R.J., Kozánek M., Wan L., Rubash H.E., Li G. (2009). Function of anterior talofibular and calcaneofibular ligaments during in-vivo motion of the ankle joint complex. J. Orthop. Surg. Res..

[44] Muc A., Gurba W. (2001). Genetic algorithms and finite element analysis in optimization of composite structures. Compos. Struct..

[45] Muc A., Muc-Wierzgoń M. (2012). An evolution strategy in structural optimization problems for plates and shells. Compos. Struct..

[46] Bukala J., Malachowski J., Szafranski T. (2016). Numerical optimization and design study of small wind turbine mast structure. Proceedings of the IECON 2016-42nd Annual Conference of the IEEE Industrial Electronics Society.

